# Lost in Green Transformation: Comment on "A Review of the Applicability of Current Green Practices in Healthcare Facilities"

**DOI:** 10.34172/ijhpm.9222

**Published:** 2025-11-17

**Authors:** Anna K.S. von Eiff, Wilfried von Eiff

**Affiliations:** ^1^Independent Researcher, Münster, Germany.; ^2^Center for Hospital Management, University of Münster, Münster, Germany.; ^3^Center for Health Care Management and Regulation, HHL Leipzig Graduate School of Management, Leipzig, Germany.; ^4^Kerckhoff Clinic, Heart-Lung Center, Bad Nauheim, Teaching Hospital of the University Clinic Gießen-Marburg, Gießen, Germany.

**Keywords:** Green Hospital Strategy, Medical Remanufacturing, Clinical Textiles, Food Services, German Healthcare Perspective, Medical Device Regulation

## Abstract

This commentary refers to the scoping review "A Review of the Applicability of Current Green Practices in Healthcare Facilities" by Soares et al and intents to bring in additional aspects influencing the green transformation in healthcare and to propose concrete means that could be helpful to move forward towards carbon footprint reduction as well as resource saving. The authors aim to help to identify how circular economy (CE) can be implemented in current hospitals. Following up on this, we would like to enrichen the discussion by three thematic areas we pose as questions: (1) Is the green transformation a prominent part of the strategic agenda of hospital? (2) What impact on the green transformation is caused by legal interventions? (3) Which concrete means help to achieve carbon footprint reduction, resource saving and have a self-financing capability? Our comment is based on practical experience with green pilot projects in German hospitals.

## Background

 The scoping review by Soares et al^1^ intents to ascertain the current status of circular economy (CE) implementation within the European Union, to reflect the applicability of CE in hospitals and to find how the application of CE to healthcare can be expanded or improved.

 Our purpose is to supplement this approach by addressing concrete examples that could offer support when implementing a green agenda step-by-step. In our opinion, the much-postulated concept of CE is a vision to date as long as the recommendations are limited on generalisations as “waste minimization,” “lasting value of resources,” and “closed loops of products within the limits of environmental, social and economic benefits.”

 Hence, our comment is on one hand focused on limitations that hinder hospital managers to meet CE requirements in pracice and on the other hand we designate concrete means that contribute to waste avoidance and resource saving with measurable success pertaining carbon footprint effects and cost containment simultaneously.

## Green Transformation – Really a Prominent Part of the Strategic Agenda?

 Studies show hospitals contribute significantly to CO_2_ emissions,^2,3^ therefore, hospitals are required to contribute to the reduction of CO_2_ emissions as well as to the saving of valuable and rare resources.

 In the chapter “Behaviour” Soares et al point out, that strategies aiming on a sustainable rollout of CE within hospitals require green awareness and willingness of individuals. We agree on this, but we recommend to additionally focuss on the pivotal role managerial decision-makers play when implementing a CE approach.

 A representative German study depicts, that despite all lip services to the necessity of a green transformation hospital managers rank the priorority of green activties surprisingly low^4^:

 “Price” is identified the most important number one decision-making criteria for purchasing and logistic managers; resource saving and impact on the environment ranks number nine.

 The decision-making criteria “Carbon Footprint Effects” is regarded as “unimportant” by 66% of the hospital procurement managers and 32% of them indicate to make purchasing decisions based on a “balance between price, product functionality, patient outcome effects and sustainability.” In day-by-day purchasing business that means, sustainability effects of medical products are very much welcome under the precondition of an attractive (low) purchasing price.

 The reason for this decision-making behaviour is easy to explain: Up to 70% of the hospitals in Germany are suffering from cost pressure and limited budgets and at least 30% report to be in the red.^5^

 Against that background, it is necessary to offer definite means and concrete recommendations that contribute to both reducing the carbon footprint and saving out-of-pocket money. Otherwise, the green transformation turns out as an illusion.

## Legal Interventions – How Do They Influence the Green Transformation?

 Legal interventions from government have to be considered when rethinking the applicability of CE. Soares et al cited the intention of the European Commission to foster a CE primarily based on waste management and policies aiming at highlighting the recycling principle and the development of material-efficient products. Beside this green supportive initiative of the European Commission it seems also necessary to look on legal settings that may hinder the realisation of green activities.

 Especially, the Medical Device Regulation requests manufacturers that new products must pass a cost-intensive and time-consuming certification process under control of a notified body. But also long-term practice-proven products are affected, exceedingly products of the classes 1s (sterile condition), 1r (reusables) and 1m (measuring function). Most of these products despite proven over years, such as venous catheters, one-way siringes and infusion tubing, have to undergo a re-certification every five years (plus post market surveillance and post-market-follow-up). The costs for certification amount to about 700k Euro for one product category. In practice, experience shows the recertification of an infusion tubing last 9 month and of a wound vacuum pump takes 18 months.^6^

 It may be expected that products with low margins will not re-certified due to cost reasons, and research budgets for the development of innovative green products tend to be reduced because the limited capital is primarily needed for re-certification. Procurement experts assume a reduction of the product portfolio offered by the manufacturers by 25% and will lead to an increase of supply chain shortages.^7^

## Green Activities – Are There Concrete Means With Self-Financing Capability Contributing to Carbon Footprint Reduction and Resource Saving?

 The questions to answer from the viewpoint of hospital managers are: “How to finance investments in sustainability programs in times of financial limitations and cost pressure?” “What concrete means are effective?” and “By which means can we achieve contributions to CO_2_ reduction and resource saving and to cost containment simultaneously?”

 In order to influence the carbon footprint extension and to contribute to resource saving already in the short-run we recommend three means with self-financing capability: Medical remanufacturing, the utilisation of reusable clinical textiles and the avoidance of waste of food.

###  Medical Remanufacturing

 Remanufacturing designates a highly specialised reprocessing technology for selected medical products, declared as single use devices (SUDs) by the manufacturer. A remanufacturing technology has to fulfil specific technological requirements proven by a “Notified Body” and has to be compliant with the European Union Medical Device Regulation (2017/245).

 A CE-marked remanufactured device must obtain the same levels of cleanliness, sterility and functionality as required of a virgin device.^8^

 The medical products eligible for re-processing (eg, ablation catheters, ultrasound scissors, urological loops) typically are expensive, are used in complex medical interventions and contain of valuable and rare resources.

 In case of a cardiac arrhythmia an ablation intervention performed with virgin SUDs (as recommended by the manufacturers) costs are estimated at €4040 compared to €2043 when using reprocessed devices (source: own research in a German heart center).

 Moreover, a remanufactured catheter has an impact of 0.87 kg CO_2_-eq/catheter compared to using a virgin catheter with an impact of 1.75 kg CO_2_-eq/catheter.^9^

 With an estimated 400 000 annual atrial fibrillation procedures in Europe the environmental impact of this procedure type is estimated equal to 84 tons CO_2_-eq emission each day.^10^

 Furthermore, it was observed that remanufacturing contributes to avoiding rationing, especially in cases where expensive devices (eg, ultrasound catheters) are used for the gentle treatment of toddlers (eg, closing a foramen ovale).^11^

###  Laundry and Clinical Textiles

 In Germany more than 70% of the hospitals prefere clinical textiles (eg, surgical drapes, theatre gowns) as SUDs; this due to lower out-of-pocket costs compared to reusable laundry. But, considering the hidden costs of SUDs and the obvious benefits of resusable textiles in terms of infection protection, wearing comfort especially during multi-hour surgical operations, and liquid absorption the price difference tends to zero.^12^

 From the ecological point of view, it should be noted that in comparison to reusable clinical textiles the disposable gownings generate a 4.5 fold higher waste volume, double the CO_2_ potential, lead to a 35% higher eutrophication potential and the summer smog potential is 110% higher.^13^ A life-cycle analysis demonstrates that the carbon footprint per reusable gown is 0.56 kg CO_2_-eq compared to 1.64 kg CO_2_-eq per the disposable product. Furthermore, the costs per use were identified 0.97 £ for disposable gowns and 0.75 £ for reusables.^14^

 In addition, disposable clinical textiles are less effective in preventing surgical site infections. The average opportunity cost for an avoidable surgical site infection episode is calculated between €4000 and €8200 added by a 10-day longer stay.^15,16^

###  Food Services

 Soares et al state hospital food services can negatively affect the environment at every stage of the food supply chain. They recommend using local food ingredients for shortening the supply chain. For hospital managers it is crucial to know, what kind of production procedure in combination with food regeneration technologies contributes to a CO_2_-eq reduction and helps saving costs by avoiding food waste simultaneously.

 Food service is responsible for 17% of all hospital emissions.^17^ The sustainability benefit of a broader offering of regional meals is uncontested. In this context it is also worth to think about a change in food components: Meat-based meals cause 3.6 kg CO_2_-eq per portion while vegetarian-based dishes are responsible for 1.7 kg CO2-eq.^18^

 More than 60% of the German hospitals have organised their food services based on the cook-and-serve principle. Such an in-house kitchen solution is energy-consuming and every day per bed up to 750 g ready-to-use prepared food gets thrown into garbage; this due to over-production (25%-30%), over-portioning (5%-15%) and other reasons.^19^

 An outsourcing analysis we recently carried out in a German heart center made transparent that it is recommendable to change food services from cook-and-serve preparation to a cook-and-freese offering, for productivity reasons as well as under the aspect of sustainability.

 In the reported case of the heart center (72 000 complete catering days per year) the daily catering costs per patient could be reduced from €23.80 to €21.70 and the daily waste of already prepared food decreased by around 300 g per catering day. A change in food production from cook & serve to cook & freeze results in 26% CO_2_-eq savings.^20^ This equals between 0.44 (vegetarian-based food) and 0.94 (meat-based food) kg CO_2_-eq per portion. Given 72 000 portions per year a carbon footprint reduction between 31 680 and 67 680 kg CO_2_-eq can be achieved.

## Conclusion

 The change from a linear to a CE in healthcare is a challenge requiring efforts from the relevant players in the field:

 Government is requested to set incentive systems that foster ecological triggered decision-making in hospitals and industry.

 Products with a high footprint emission could be banned from payment in the diagnosis-related group reimbursement system or could burdened with a special tax.

 Manufacturers could be obliged to offer a product portfolio that contains a given quota of products especially designed for medical remanufacturing and for a high number of repair cycles.

 Manufacturers are requested to offer more repairable and reusable products. Cooperations between manufacturers and reprocessing service providers drive the development of limited patient use products. That means in effect, devices with a guaranteed number of safe and functionality-proven reprocessing cycles.^21^ This requirement is in accordance with Soares et al and their suggestion to reduce healthcare waste through the replacement of single-use devices with reusable ones and the design of reusable products to replace the disposable ones.

 A sustainability strategy isolated triggered by an ecological purpose may have the potential of harming the patient or lead to a detoriation of the medical quality. Both effects are questionable in terms of ethics. For eyample, magnetic resonance imaging (MRI) and computed tomography (CT) are energy-consuming technologies. The mean admission of one MRI diagnosis is 17.5 kg CO_2_-eq and of one CT is 9.2 kg CO_2_-eq.^22^ Any sustainabilitity-motivated phase-down must safeguard diagnostic yield and patient outcomes; stepwise pilots with controlled by clinical key performance indicators are required.

## Limitations

 Our comment is based on research initiatives and own practical experience referring to the situation in the German healthcare system. Hence, the transferability of our findings and recommendations to other European countries may be limited, this due to different reimbursement systems, legal frameworks, health insurance approaches and incentives for a green decision-making in the C-suite of hospitals.

## Disclosure of artificial intelligence (AI) use

 Not applicable.

## Ethical issues

 Not applicable.

## Conflicts of interest

 Authors declare that they have no conflicts of interest.
